# The cortisol stress response following surgery for proximal femur fractures in geriatric patients – a prospective pilot study

**DOI:** 10.3389/fendo.2026.1820534

**Published:** 2026-05-07

**Authors:** Maximilian M. Menger, Laura E. Streck, Benedikt J. Braun, Steven C. Herath, Christof K. Audretsch, Maximilian Bamberg, Michael D. Menger, Tina Histing, Johann Fontana

**Affiliations:** 1 Department of Trauma and Reconstructive Surgery, BG Trauma Center Tuebingen, Eberhard Karls University Tuebingen, Tuebingen, Germany; 2 Department of Anesthesiology and Intensive Care Medicine, BG Trauma Center Tuebingen, Tuebingen, Germany; 3 Institute for Clinical and Experimental Surgery, Saarland University, Homburg, Germany; 4 Medical Faculty Mannheim, Ruprecht Karls University Heidelberg, Mannheim, Germany

**Keywords:** cortisol, geriatric, proximal femur fracture, surgery, trauma, interleukin-6

## Abstract

**Introduction:**

Aging impairs the suprachiasmatic nucleus function, compromising the adrenocortical circadian rhythmicity and, potentially, affecting the cortisol stress response. Relative cortisol insufficiency may contribute to peri- and postoperative complications and increase mortality. While proximal femoral fractures in geriatric patients are among the most common musculoskeletal injuries, little is known about the adrenal stress response in geriatric patients undergoing surgery for this injury.

**Methods:**

The present prospective pilot study investigated the postoperative cortisol levels in 20 geriatric patients (age > 70 years) with proximal femur fractures who were admitted to the intensive care unit (ICU) of a level I trauma center. Cortisol levels were obtained by blood sampling after surgery in conjunction with daily routine laboratory examinations. Further data acquisitions included demographic information as well as surgery and ICU stay related characteristics.

**Results:**

The results showed that 35% of all patients demonstrated cortisol levels even below the basal threshold of 276 nmol/L, which is commonly used for the definition of critical illness-related corticosteroid insufficiency (CIRCI). Correlation analysis revealed a significant association between interleukin-6 (IL-6) and cortisol levels, whereas procalcitonin (PCT), leukocyte count and C-reactive protein (CRP) had no predictive value. A considerable proportion of geriatric trauma patients suffering from proximal femoral fractures admitted to the ICU exhibited lower-than-expected cortisol levels.

**Conclusion:**

Postoperative determination of cortisol levels should be considered in geriatric patients undergoing postoperative monitoring. This would allow to identify patients with an attenuated cortisol stress response and enable timely initiation of treatment, such as probatory administration of hydrocortisone, to improve patient outcome. Moreover, IL-6 may represent a potential marker for the monitoring of the postoperative endocrine stress response in geriatric trauma patients.

## Introduction

Proximal femur fractures are among the most common injuries in trauma and orthopedic surgery, mostly affecting geriatric patients with an age of 75 years or older. With a projected increasing geriatric population over the next decades, the incidence of these injuries will likely rise accordingly (1). In 1990, approximately 1.3 million proximal femur fractures were reported worldwide (2), whereas by 2050 the number is estimated to range between 7.3 and 21.3 million (1, 3).

For geriatric patients, proximal femur fractures often represent an event with a significant impact on their self-sustainability and everyday life. Only 40 to 60% of these patients regain their pre-injury level of activity and mobility (4). Thus, a considerable number of patients require extensive aftercare, which not only places a significant burden on the individuals and their families, but also imposes considerable demands on the healthcare system. Moreover, the elderly suffer from comorbidities and frailty, which increases the vulnerability to stressors due to a decline in the reserve of various physiological systems (5). This, in turn, may contribute to one-year mortality rates in geriatric patients suffering from proximal femur fractures as high as 40% (6).

Aging may lead to an altered endocrine response to trauma and surgery, including a dysregulation of the cortisol release. Following surgical trauma, cortisol plays a central role in regulating the inflammatory response by exerting anti-inflammatory actions, raising blood glucose levels, stabilizing cellular membranes, and increasing blood pressure (7, 8). There is evidence that aging impairs the suprachiasmatic nucleus function, including the hippocampus. Thus, its ability to regulate adrenocortical circadian rhythmicity and stress response is compromised, resulting in a reduced circadian fluctuation of cortisol secretion (9, 10) and potentially affecting the cortisol release after surgery. Relative cortisol insufficiency can present with a variety of symptoms including hypotension resistant to fluid therapy, electrolyte imbalance, fatigue, nausea, loss of appetite as well as alterations of the mental status (11). All of these may lead to intensive care unit (ICU) admission and eventually compromise the patient outcome (12). Due to relevant cortisol deficits of up to 20% and even higher incidences among patients with septic shock, the relevance of an acute adrenal insufficiency in ICU patients is of increasing concern (13). Accordingly, the Society of Critical Care Medicine (SCCM) defined guidelines for a critical illness-related corticosteroid insufficiency (CIRCI) (14). This pathology is characterized by an impairment of the hypothalamic−pituitary axis stress response function during critical illness. These guidelines recommend the supplementary use of glucocorticoids in specific settings, such as acute respiratory distress syndrome, sepsis, community-acquired pneumonia and cardiac surgery (15, 16).

However, there is no information on alterations of cortisol levels in geriatric patients who require ICU-admission following surgery for proximal femur fractures and their significance for patient outcome. Thus, corresponding guidelines and treatment concepts are lacking. The present study was designed to reduce this data gap by prospectively analyzing the postoperative cortisol levels in geriatric patients undergoing surgical treatment for proximal femur fractures.

## Materials and methods

### Ethics and patient recruitment

The present study received approval from the Institutional Review Board (reference number: 464/2023BO2). All participating patients (n = 20) were admitted to the ICU of a level I trauma center between 09/2023 and 08/2024 following surgery for proximal femur fractures. Inclusion criteria were geriatric trauma patients over 70 years of age undergoing surgical treatment for proximal femur fractures and postoperative admission to the ICU. Patients with long-term corticosteroid therapy and pituitary tumors were excluded. The participation of the patients was confirmed by written consent. Cortisol levels were determined within 24 hours after surgery in combination with the daily routine laboratory examinations. To prevent inaccuracies due to the circadian fluctuation of cortisol, all blood samples were drawn between 5 and 6 a.m.

### Data acquisition

**Figure 1:**
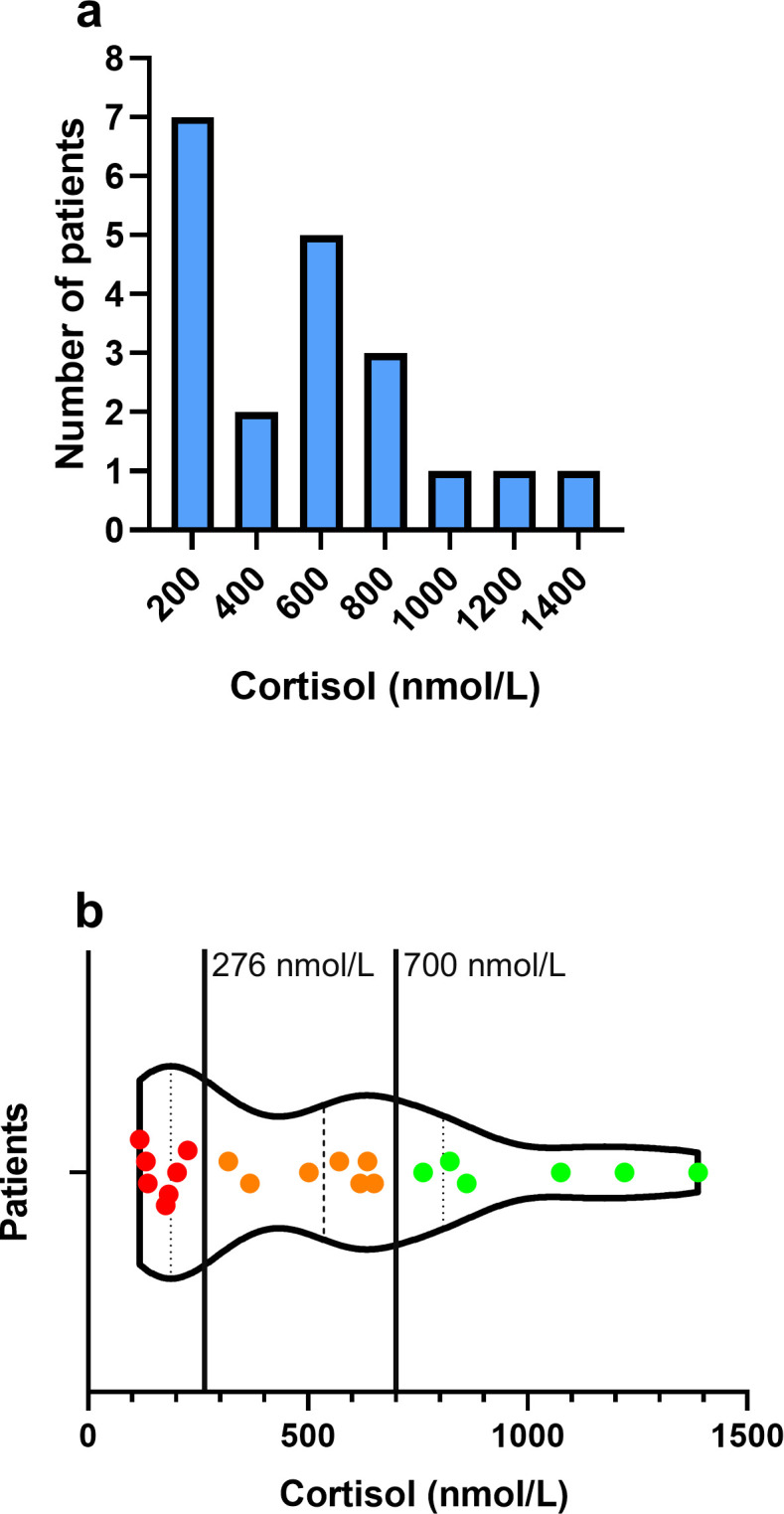
Quantification of cortisol serum levels. **(a)** Histogram of cortisol serum levels (nmol/L) of geriatric trauma patients suffering from proximal femoral fractures after surgery. The bin centers (200, 400, 600, 800, 1000, 1200 and 1400 nmol/L) are indicated, with a respective bin width of 200 nmol/L. **(b)** Violin plot demonstrating the distribution of serum cortisol levels (nmol/L) in context to expected cortisol levels after elective musculoskeletal surgery or moderate to highly invasive surgical procedures (700 nmol/L) and the upper cortisol level for the definition of CIRCI (276 nmol/L). Green circles indicate patients with cortisol levels ≥ 700 nmol/L, orange circles indicate patients with cortisol levels < 700 nmol/L and ≥ 276 nmol/L and red circles indicate patients with cortisol levels < 276 nmol/L.

Demographic parameters were assessed prior to surgery and included: age, sex, Body Mass Index (BMI) and the American Society of Anesthesiology (ASA) score. Moreover, the type of surgical procedure, reason for ICU-admission, length of ICU-stay, duration of surgery, time from admission to surgery, time between wound closure and blood sampling and demand of vasopressor (norepinephrine) therapy were assessed. Parameters analyzed by blood sampling included serum cortisol, interleukin-6 (IL-6), procalcitonin (PCT), leukocyte count and C-reactive protein (CRP) within 24 hours after surgery. Serum cortisol levels are displayed as histogram and as violin plot. The expected postoperative cortisol levels in aged patients undergoing elective musculoskeletal surgery or patients after moderately and highly invasive surgery (≈ 700 nmol/L) (10, 17) as well as basal cortisol levels (≈ 276 nmol/L) for the diagnosis of CIRCI (14, 15) are indicated (**Figures 1a, b**). Cortisol serum levels were determined by a competitive immunoassay based on immunoluminescence (Atellica^®^ IM analyzer, Siemens Healthcare GmbH, Erlangen, Germany). The limit of detection (LoD) corresponded to 3.86 nmol/L.

### Statistical analysis

Descriptive analysis is presented as mean and standard deviation (SD) for continuous variables, and numbers and percentage for categorical variables. Due to the sample size, a conservative approach using non-parametric statistical tests was taken. Spearman’s rank correlation coefficient (*ρ*) was used to evaluate the association between cortisol levels, inflammatory parameters (IL-6, leukocytes, CRP, PCT), age, length of ICU-stay, time from admission to surgery, duration of surgery, time between wound closure and blood sampling as well as demand of vasopressor (norepinephrine) therapy. Statistical significance was set at *p* < 0.05. Statistical analysis was performed with SigmaPlot 13.0 software (Jandel Corporation, San Rafael, CA, USA), GraphPad Prism software version 8.0 (GraphPad Software, La Jolla, CA, USA) and JASP software version 0.95.2 (JASP Team, Amsterdam, the Netherlands).

## Results

### Patients’ demographics

The included patient cohort (n = 20) showed a mean age of 87.6 years, representing a geriatric population. Moreover, the data demonstrates that most patients (70%) had an ASA-score of 3 and higher, indicating a variety of comorbidities and associated frailty. The majority of patients were female (75%). The exact data are presented in **Table 1**.

**Table 1:** Data of patients’ demographics at the time of surgery.

Age	87.6 ± 6.7 (years)
BMI	25.1 ± 5.7 (kg/m²)
ASA	
1 2 3 4	0/20 (0%) 6/20 (30%) 13/20 (65%) 1/20 (5%)
Sex	
Male Female	5/20 (25%) 15/20 (75%)

ASA score and sex are indicated as number of patients/total number of patients (percentage).

### Characteristics of surgery and ICU stay

The included patients suffering from proximal femoral fractures were treated with either hemiarthroplasty (n = 8), femoral nailing (n = 11) or femoral stem revision (n = 1) depending on the fracture morphology. The majority of patients were admitted to ICU due to circulatory insufficiency (n = 14). Other reasons for ICU-admission included respiratory insufficiency (n = 2), altered mental status (n = 2), circulatory insufficiency and altered mental status (n = 1) as well as electrolyte imbalance (n = 1). Of note, the time between hospital admission to surgery varied between the patients, most likely due to the profile of comorbidities, medication and type of surgery. Nevertheless, most patients (n = 13) were treated within the first 24 hours after hospitalization. Blood samples were drawn on the first postoperative day with a mean of 573.4 minutes (= 9.6 hours) after the wound closure. Perioperative vasopressor therapy was necessary in most patients (n = 18), whereas only n = 3 patients were still in need of vasopressor therapy on the first post-surgical day. Accordingly, the overall duration of the ICU stay was rather short, with a mean of 2.4 days. The exact data are presented in **Table 2**.

**Table 2:** Data of surgery and ICU-hospitalization characteristics.

Type of surgery	
Hemiarthroplasty	8/20 (40%)
Femoral nailing	11/20 (55%)
Femoral stem revision	1/20 (5%)
Reason for ICU admission	
Respiratory insufficiencyElectrolyte imbalance	2/20 (10%)
Circulatory insufficiency	14/20 (70%)
Altered mental status	2/20 (10%)
Circulatory insufficiency and altered mental status	1/20 (5%)
Electrolyte imbalance	1/20 (5%)
Hospitalization characteristics	
Time between hospital admission to surgery	1380.1 ± 1457.3 min
Duration of surgery	86.5 ± 38.7 min
Time between wound closure and blood sampling	573.4 ± 271.9 min
Intraoperative vasopressor therapy	0.04 ± 0.05 µg/kg/min
Vasopressor therapy on day 1 at ICU	0.01 ± 0.02 µg/kg/min
Length of ICU-stay	2.4 ± 2.1 days

The type of surgery and reason for ICU admission are indicated as number of patients/total number of patients (percentage).

### Cortisol levels and correlation analysis

**Figure 2:**
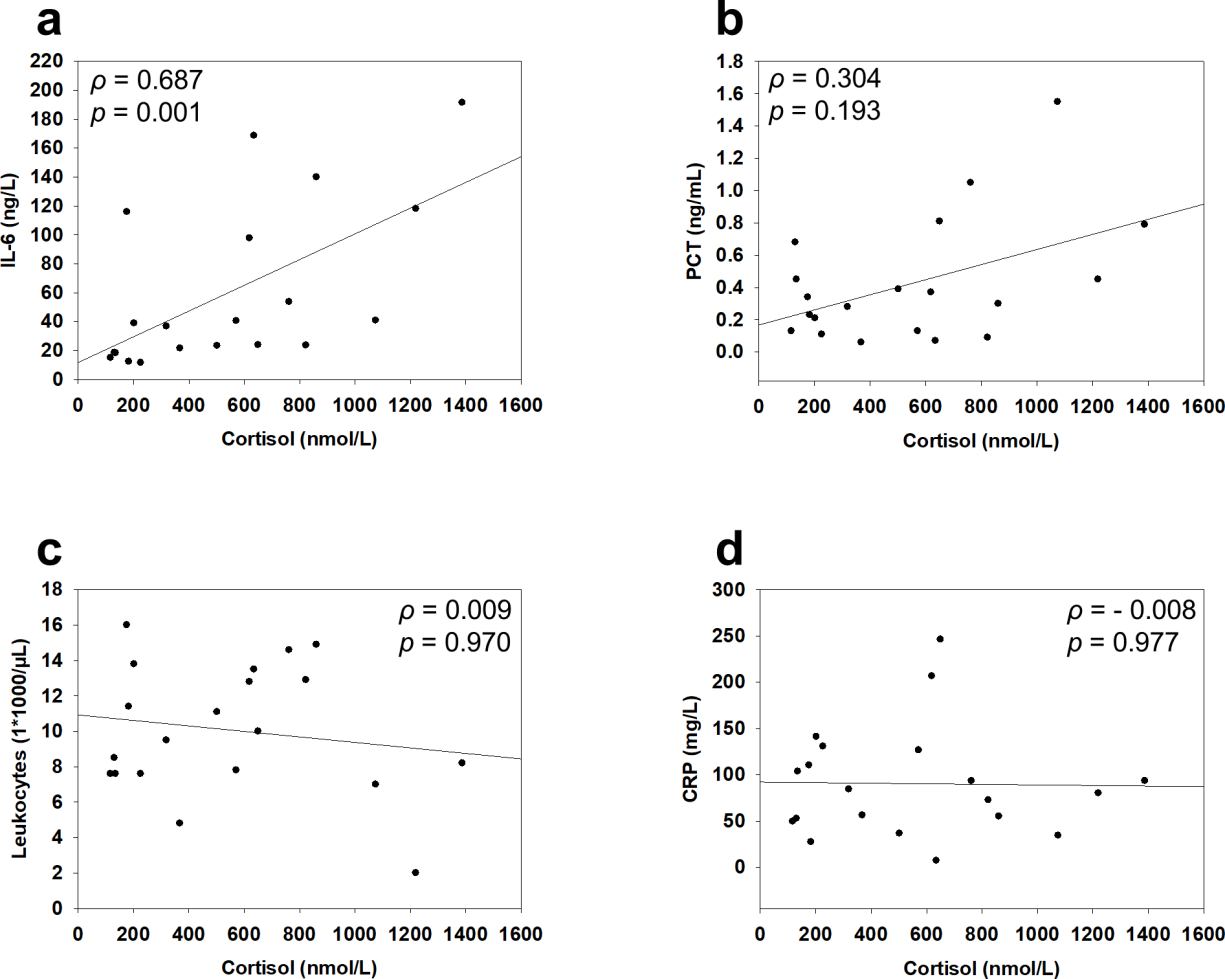
Correlation analysis between cortisol serum levels and inflammatory parameters. **(a-d)** Correlation analysis between cortisol serum levels and the inflammatory parameters IL-6 (ng/L), PCT (ng/mL), leukocytes (1*1000/µL) and CRP (mg/L). Corresponding Spearman’s ρ and *p* values are demonstrated.

The cortisol levels obtained demonstrated a wide range between individual patients (range: 117–1388 nmol/L; mean ± SD: 548.2 ± 383.2 nmol/L) (**Figures 1a, b**). Interestingly, the majority of patients (n = 14, 70%) exhibited cortisol levels below 700 nmol/L, whereas values following elective musculoskeletal surgery or lower limb trauma are typically above this threshold (10, 18). Moreover, some patients (n = 7, 35%) even demonstrated cortisol levels below the threshold of CIRCI definition (276 nmol/L) (**Figure 1b**). Correlation analysis with inflammatory parameters revealed a significant correlation between IL-6 and cortisol levels (**Figure 2a**). However, no significant correlation was found between the other inflammatory parameters PCT, leukocyte count and CRP (**Figures 2b–d**).

Additional correlation analysis between cortisol levels and patients’ age as well as surgery and hospitalization characteristics demonstrated no significant association for age, length of ICU-stay, time of admission to surgery, duration of surgery, time between wound closure and blood sampling, and demand of vasopressor therapy (data are shown in **Table 3**).

**Table 3:** Correlation analysis between cortisol levels and patients’ age as well as surgery and hospitalization characteristics.

Variable	Spearman’s *ρ*	*p*
Cortisol–Age	0.191	0.421
Cortisol–Duration of surgery	0.043	0.858
Cortisol–Time between hospital admission to surgery	0.347	0.134
Cortisol–Time between wound closure and blood sampling	-0.161	0.496
Cortisol–Perioperative vasopressor therapy	0.117	0.624
Cortisol–Vasopressor therapy on day 1 at ICU	0.314	0.177
Cortisol–Length of ICU-stay	0.013	0.956

Corresponding Spearman’s *ρ* and *p* values are demonstrated.

## Discussion

The present study characterizes, for the first time, the early postoperative cortisol stress response in geriatric patients requiring ICU admission after surgery for proximal femur fractures. Interestingly, 35% of all these patients demonstrated cortisol levels below the basal threshold of 276 nmol/L, which is commonly used for the diagnosis of CIRCI (15), indicating an attenuated cortisol stress response after trauma and subsequent surgery.

The exact pathophysiology resulting in an inadequate cortisol release and the associated dysregulated systemic inflammation is not yet completely understood. Insufficient cortisol release can result in a variety of symptoms including circulatory and respiratory insufficiency as well as fatigue, nausea, loss of appetite and altered mental status (14, 19). The established guidelines of the SCCM recommend a diagnosis of CIRCI by a change in total serum cortisol of < 9 μg/dl (248 nmol/L) after cosyntropin (250 μg) administration or a random total cortisol value of < 10 μg/dl (276 nmol/L) (15). Notably, the evaluation of random total cortisol for the diagnosis of such an endocrine deficit in emergency trauma patients represents a more feasible approach than performing dynamic testing with additional cosyntropin administration. In the acute trauma setting, patients often require rapid diagnostic assessment and immediate clinical decision-making, which limits the practicality of time-consuming stimulation tests. Therefore, the measurement of random total cortisol levels may serve as a more pragmatic diagnostic tool to identify patients with an insufficient cortisol stress response. While these numbers define an absolute corticosteroid deficit, there is little information on values defining relatively critical cortisol levels in specific diseases, especially with regard to geriatric trauma patients.

However, some studies report on the cortisol levels after lower limb trauma and surgical procedures in relation to their invasiveness. Jain et al. (18) analyzed the cortical stress response in patients suffering from lower limb trauma and found cortisol levels exceeding 700 nmol/L, 24 hours after surgery in patients receiving epidural as well as standard analgesia procedures. In line with these findings, Prete et al. (17) demonstrated that cortisol levels typically rise above 700 nmol/L within 24 hours after moderately and highly invasive surgery. Interestingly, the authors revealed that aged patients (> 60 years) exhibit higher cortisol concentrations during the postoperative period when compared to the younger cohort (≤ 60 years). Nevertheless, those findings remain controversial. Zhong et al. (10) investigated the cortisol levels after elective total hip arthroplasty, and reported findings that contradict those of Prete et al. (17). Specifically, the authors found significantly lower cortisol levels (701.85 ± 156.32 (mean ± SD)) in aged patients (> 65 years) after elective total hip arthroplasty when compared to the middle-aged patient collective (40–65 years; 807.12 ± 161.34) (10). It should be considered that the cortisol stress response varies significantly depending on the type of anesthesia and analgesia (20) as well as patient-related factors such as gender and age (17). Moreover, in this specific geriatric trauma population, the currently available data may only insufficiently reflect the physiology of the frail elderly, whose baseline HPA-axis function is already altered. Hence, further research is warranted to determine a definite threshold for expected cortisol levels following musculoskeletal trauma and surgery, considering patient-related characteristics.

Notably, the age-related alterations in the hypothalamic-pituitary-adrenal (HPA) axis are complex and yet not completely understood. Apparently, the aging population demonstrates an overall increase in cortisol secretion, accompanied by a disruption of the negative cortisol feedback loop, and an attenuation of the cortisol diurnal pattern (21). However, reduced feedback mechanisms may also compromise the cortisol stress response in geriatric patients. Studies investigating the differences in the cortisol release between adult and elderly individuals after psychosocial challenge and stress have demonstrated inconclusive results (21–23). Hence, further research is needed to fully elucidate the mechanisms underlying the cortisol stress response after trauma and surgery across different age groups. The impaired HPA-axis function, characterized by an attenuated circadian rhythmicity, reduced dexamethasone suppression, and a diminished response to an adrenocorticotropic hormone (ACTH) stimulation, suggest that the functional reserve of the adrenal cortex is already compromised before the trauma occurs (24). The fracture (first hit) might already deplete the functional reserve of the adrenal cortex. This is indicated by the finding that elderly women suffering from proximal femur fractures demonstrate a reduced incremental ACTH and cortisol response after corticotropin-releasing hormone (CRH) (25). Consequently, the adrenal reserve may be insufficient to meet the physiological demands of the subsequent surgery (second hit).

Taking these findings into account, a considerable number of geriatric patients in the present study demonstrated a relative cortisol deficiency during the early postoperative period with cortisol levels below 700 nmol/L. It may be hypothesized that the second hit of the surgical procedure following only a few hours after the initial trauma, exceeds the already compromised capacity of the aged organism to generate an adequate stress response. This inadequate endocrine stress response may have detrimental effects on the patients’ outcome and contribute to the observed symptoms that led to ICU admission such as circulatory and respiratory insufficiency as well as electrolyte imbalance and altered mental status. In line with this assumption Kwok et al. (26) revealed in a prospective study involving 189 trauma patients, that patients with severely low cortisol levels (< 414 nmol/L) on admission demonstrated higher blood requirements, vasopressor use and a higher mortality rate. Interestingly, Nicholson et al. (27) found cortisol levels below 414 nmol/L for 72 hours after surgery in patients undergoing major pelvic reconstructive surgery. Notably, acetabular and pelvic fractures often require extensive surgical approaches, such as the anterior ilioinguinal or the posterior Kocher-Langenbeck approach (28, 29). Hence, the observed low cortisol levels by Nicholson et al. (27) may represent an insufficient cortisol release, resulting in a relative cortisol insufficiency due to the largely invasive surgery.

Perioperative glucocorticoid replacement, such as a single dose of hydrocortisone, may ameliorate the negative impacts of relative cortisol insufficiency. The perioperative administration of glucocorticoids has been discussed throughout the literature due to potential side effects, including surgical site infection, delayed wound healing and dysregulation of blood glucose levels. However, current evidence indicates that a single dose of glucocorticoids does not increase the risk for postoperative surgical site infection (30, 31). Nevertheless, the impact on delayed wound healing should be interpreted with caution due to its imprecise definition in the literature (30, 31). Moreover, although the increase in blood glucose levels after glucocorticoid administration is apparent, it is in most cases not clinically relevant (32, 33). Therefore, the existing literature indicates that a single dose administration of hydrocortisone does not result in a significant increase of perioperative complications. Of note, although geriatric patients were included in these studies, a specific analysis of perioperative glucocorticoid-associated side effects in a geriatric subpopulation are missing. Hence, future research is warranted to address this gap of knowledge.

The correlation analysis demonstrated a significant positive correlation between cortisol levels and IL-6, but not PCT, leukocyte count or CRP. Interestingly, Bjornsson et al. (34) reported similar findings by analyzing the inflammatory and adrenal response in patients undergoing hip arthroplasty, and found a significant correlation between cortisol secretion and IL-6. These results are also consistent with our previous study, which revealed a significant correlation between cortisol levels and IL-6 in patients suffering from burn injury (35). Thus, in contrast to PCT, leukocyte count and CRP, IL-6 might represent a potential marker to monitor the endocrine stress response in geriatric trauma patients. The pro-inflammatory cytokine IL-6 has been recognized as potent stimulator of the HPA-axis, thereby stimulating the release of CRH and ACTH, and subsequently increasing cortisol levels (36, 37). However, during physiological stress IL-6 can also directly stimulate cortisol secretion, HPA-independent, by binding to its receptors on the adrenal gland (38). This may lead to a maladaptive response of the HPA-axis, also referred to as ACTH-cortisol dissociation, characterized by the absence of an increased pituitary secretion of ACTH. ACTH–cortisol dissociation plays a major role in the pathogenesis of critical illness and sepsis, and is also associated with postoperative complications (39, 40). Hence, increased cortisol levels may not necessarily conclude an appropriate stress response and superior patient outcome. By additional endocrinological analysis such as CRH and ACTH measurement, future studies might elucidate if the observed correlation of IL-6 and cortisol in geriatric trauma patients is the result of a physiological stimulation of the HPA-axis, or the result of an ACTH-cortisol dissociation leading to a maladaptive endocrine response.

The conclusion of the present prospective study is limited by the small cohort of 20 patients and, therefore, provides only preliminary data. This may explain the lack of statistically significant correlations between cortisol levels and patients’ age, as well as surgery and hospitalization characteristics, such as duration of surgery, vasopressor therapy and length of ICU stay. Moreover, it cannot be excluded that the attenuated cortisol stress response observed in the present study may be due to patient-related factors rather than trauma and surgery. Hence, this study should be considered as an exploratory pilot study, for subsequent prospective studies. These future clinical trials require larger and homogenous patient cohorts as well as preoperative values and an extended follow-up period to further elucidate the pathophysiological mechanisms underlying the insufficient cortisol response in geriatric trauma patients. In particular, these studies should focus on patient-related factors such as comorbidities and medications, as well as perioperative and hospitalization characteristics. The type of surgical treatment and the timing of blood sample collection relative to surgery may be likely critical determinants of postoperative cortisol levels and should be carefully standardized and evaluated. In addition, a broader endocrine assessment, including the analysis of cortisone and dehydroepiandrosterone (DHEA) would contribute to a thorough characterization of the hormonal stress response after trauma and surgery.

Taken together, the present study revealed that a considerable proportion of geriatric patients exhibited a potentially relevant attenuation of the cortisol stress response after trauma surgery. Hence, the determination of cortisol levels should be considered in geriatric patients presenting with unspecific circulatory, respiratory, or neurological symptoms on the first postoperative day. Furthermore, IL-6, in addition to a random total cortisol level, may represent a potential marker for the monitoring of the postoperative endocrine stress response in geriatric trauma patients. The clinical impact of a relative cortisol deficit needs to be elucidated in further studies with larger patient cohorts. Nevertheless, the application of a probatory hydrocortisone dose seems reasonable in geriatric trauma patients with unspecific post-surgical symptoms like fatigue, altered mental status or orthostatic hypotension.

## Data Availability

The raw data supporting the conclusions of this article will be made available by the authors upon reasonable request.
